# Predictors of Gestational Weight Gain among White and Latina Women and Associations with Birth Weight

**DOI:** 10.1155/2016/8984928

**Published:** 2016-09-04

**Authors:** Milagros C. Rosal, Monica L. Wang, Tiffany A. Moore Simas, Jamie S. Bodenlos, Sybil L. Crawford, Katherine Leung, Heather Z. Sankey

**Affiliations:** ^1^Department of Medicine, Division of Preventive and Behavioral Medicine, University of Massachusetts Medical School, 55 Lake Avenue North, Worcester, MA 01655, USA; ^2^Boston University School of Public Health, 801 Massachusetts Avenue, Boston, MA 02215, USA; ^3^Harvard School of Public Health, 677 Huntington Avenue, Boston, MA 02215, USA; ^4^Department of Psychology, Hobart and William Smith Colleges, 217 Gulick Hall, Geneva, NY 14456, USA; ^5^Department of Obstetrics and Gynecology, Baystate Medical Center, 759 Chestnut Street, Springfield, MA 01199, USA

## Abstract

This study examined racial/ethnic differences in gestational weight gain (GWG) predictors and association of first-trimester GWG to overall GWG among 271 White women and 300 Latina women. Rates of within-guideline GWG were higher among Latinas than among Whites (28.7% versus 24.4%, *p* < 0.016). Adjusted odds of above-guideline GWG were higher among prepregnancy overweight (OR = 3.4, CI = 1.8–6.5) and obese (OR = 4.5, CI = 2.3–9.0) women than among healthy weight women and among women with above-guideline first-trimester GWG than among those with within-guideline first-trimester GWG (OR = 4.9, CI = 2.8–8.8). GWG was positively associated with neonate birth size (*p* < 0.001). Interventions targeting prepregnancy overweight or obese women and those with excessive first-trimester GWG are needed.

## 1. Introduction

Significant evidence ties gestational weight gain (GWG) to short- and long-term maternal and infant outcomes. To optimize maternal and child health, the Institute of Medicine (IOM) provides guidelines for GWG based on prepregnancy body mass index (BMI) [1]. Greater GWG is recommended for women with prepregnancy BMIs in the underweight (28–40 pounds (lbs), 12.7–18.1 kg) or healthy weight (25–35 lbs, 11.3–15.9 kg) range, with less GWG recommended for prepregnancy overweight (15–25 lbs, 6.8–11.3 kg) and obese (11–20 lbs, 5.0–9.1 kg) women. However, only 22 to 40% of women attain GWG within the recommended ranges [2–8], and women of lower socioeconomic status and racial/ethnic minority women have lower adherence to GWG guidelines [5, 9–11]. Among Latina women and depending on national origin, estimates of excessive GWG range from 36 to 51%, whereas estimates of insufficient GWG range from 17 to 30% [7, 9, 10, 12, 13].

Socioeconomic and racial/ethnic disparities in achieving recommended GWG are further compounded by higher pregnancy rates and greater odds of adverse birth-related outcomes among socioeconomically disadvantaged and racial/ethnic minority populations than their more affluent and White counterparts. The pregnancy rate of Latina women in the US is estimated to be two-thirds higher than that of non-Latino Whites [14]. Within the Latina population, nearly half of Caribbean Latina women experience GWG above IOM guidelines [9], and Puerto Rican Latinas are among women with the highest rates of low birth weight neonates [15] and preterm births [16], both predictors of infant mortality [17]. However, little is known about why adherence to guidelines is low among this population. Identifying and understanding factors driving racial/ethnic differences in GWG are a priority to target maternal and child health disparities in this growing and at-risk population.

Given the numerous adverse health consequences of excessive and insufficient GWG for the mother and the offspring [1, 4, 18–21], understanding the risk factors for low adherence to IOM-recommended GWG and intervening in at-risk groups are of utmost importance. In targeting interventions, timing of GWG may be important. However, little is known about the influence of early GWG (e.g., first trimester) on overall GWG and other maternal and infant outcomes. A prospective study of a predominantly White female sample indicated that maternal weight change in the first trimester was a stronger predictor of birth weight than weight change in the second or third trimester [22]. However, research on early GWG among Latina women is lacking. The timing and extent of GWG may also be an important determinant of birth weight as well as other maternal and prenatal outcomes; thus, early identification of women who are at risk of excessive or inadequate GWG may be critical to guide the timing and content for intervention delivery to maximize maternal and prenatal health and reduce health disparities.

To address gaps in the literature, this study aimed to examine differences in predictors of gestational weight gain (GWG), assess the association of first-trimester GWG to overall GWG between non-Latina White and Latina women, and examine GWG status with birth outcomes. We hypothesized that women who were overweight or obese before pregnancy would have higher odds of GWG outside of IOM recommendations and that first-trimester GWG status (below, within, or above guideline) would positively correlate with overall GWG.

## 2. Methods

### 2.1. Participants and Setting

The study's targeted population included non-Latina White and Latina women who received prenatal care from private providers and hospital clinics (i.e., a resident clinic and a midwifery clinic). The study was conducted at Baystate Medical Center, a large tertiary care facility in western Massachusetts with an average of 4,300 deliveries each year, approximately 57% of them to Latina women (primarily of Puerto Rican origin).

### 2.2. Procedures

Identification of participants included two screening steps. First, electronic medical record database searches were performed for a retrospective cohort of women who had live deliveries (preterm or full-term) at the medical center from September 1, 2005, to August 31, 2006. Women with multifetal pregnancies, unknown ethnicity, and primary language other than English or Spanish were excluded. A total of 3,966 (of 4,300) patient records met these criteria. Based on estimates of adherence to IOM guidelines in other samples, a sample size of at least 400 women was required for adequate power analysis for the current study. Thus, the second screening step consisted of randomly selecting one quarter (*n* = 1,016) of eligible patient records, stratified by ethnicity (non-Latina White and Latina) and site of prenatal care (hospital clinics and private providers), for additional participant eligibility screening via paper medical chart review. A total of 445 records were excluded. Reasons for exclusion included missing data on prepregnancy weight (*n* = 226) or height (*n* = 4), missing dates of prenatal measurements (*n* = 138), no documentation of prenatal visits in the first trimester of pregnancy (*n* = 296), maternal history of gastric bypass (*n* = 2), or maternal diagnosis of pregestational diabetes (*n* = 31). Of excluded records, 60% were excluded for one criterion and 40% were excluded for two or more criteria.

A scannable medical record abstraction form was developed by the research team. The form included fields for recording participant demographics (date of birth, race/ethnicity, primary language, marital status, insurance type, parity, and employment status), psychiatric history (i.e., documented psychiatric diagnosis or use of psychiatric medication), height, and dates and measured weights at each prenatal visit. Three research assistants were trained in the process of data abstraction from paper medical records until 100% interrater reliability was achieved. Data from completed and cross-checked abstraction forms were scanned and were uploaded into a SAS database.

Data abstraction was performed from 2007 to 2008. During this time frame, revisions of IOM's GWG guidelines were anticipated and were available following data cleaning procedures and at the time of analyses. Thus, the investigative team decided* a priori* to utilize 2009 guidelines [1] in categorizing GWG measures (described below) with the goal of providing an estimate of likely nonadherence to new recommendations and associated outcomes. Additionally, the 2009 guidelines did not differ greatly from former guidelines yet offered the benefit of a recommended range of gain for obese women in contrast to the previously stated “at least 15 pounds (6.8 kg)” without an upper bound [17]. All study protocols and procedures were approved by the Baystate Medical Center Institutional Review Board and the University of Massachusetts Medical School Institutional Review Board.

### 2.3. GWG Measures

Height and prepregnancy weight were obtained from prenatal forms in participants' medical records. Customarily, height is measured by obstetric provider office staff and prepregnancy weight is self-reported by pregnant women at their first prenatal appointment. Prepregnancy BMI was calculated as weight (kg)/height squared (in meters) and categorized as follows: underweight (BMI < 18.5 kg/m^2^); healthy weight (18.5 kg/m^2^ ≤ BMI < 25 kg/m^2^); overweight (25 kg/m^2^ ≤ BMI < 30 kg/m^2^); and obese (30 kg/m^2^ ≤ BMI) [17, 23].

Gestational weight measures were routinely obtained by clinical staff as part of standard obstetric care appointments, as is customary. At each visit, women are weighed and their weight is recorded in prenatal health records, along with gestational age. Each participant's GWG status was determined based on prepregnancy BMI, gestational age, and weight gain at the time of the weight measure. For each prepregnancy weight status category, IOM-recommended trajectories of weight gain were defined (1) in terms of minimum and maximum total weight gain at week 13 (end of first trimester) and (2) for subsequent weeks in terms of minimum and maximum weight gain per week. Thus, for each week of gestational age, a minimum and maximum recommended weight gain were calculated.

First-trimester GWG status was determined using the last weight measure recorded during the first trimester. GWG status in the first trimester was assessed by comparing first-trimester GWG (calculated by subtracting pregravid weight from weight at the last first-trimester prenatal visit) to the IOM-recommended GWG range for gestational age at the last first-trimester prenatal visit. Similarly, GWG status at delivery was determined using weight measured from the last recorded prenatal appointment and was assessed by comparing total GWG (calculated by subtracting pregravid weight from weight at the last prenatal visit prior to delivery) to the IOM-recommended GWG range for gestational age at the last prenatal visit (the average period between the last prenatal visit and delivery is estimated at 6.6 days) [24]. GWG status was categorized as follows: inadequate or “below” if weight gain for gestational age was below the lowest value of the recommended range; appropriate or “within” if weight gain for gestational age was between the recommended range lowest and highest values; and excessive or “above” if weight gain for gestational age was above the highest value of the recommended range.

### 2.4. Outcome Measures

Gestational age at delivery was calculated based on best dates for estimated date of confinement (EDC). EDC is determined as per clinician evaluation considering concordance of the last menstrual period and first-trimester ultrasound [25] and documented on the medical record based on clinical care standards. Pregnancies delivered at < 37 weeks were categorized as preterm and those delivered at ≥ 37 weeks were full term. Neonate birth weight recorded by nursing staff at the time of delivery was abstracted from the inpatient record. Neonates were categorized as small for gestational age (SGA) and large for gestational age (LGA) if birth weight was <10th and ≥90th percentile, respectively, of 1999-2000 US national reference data for singleton gestations, accounting for gestational age and gender [26, 27]. Regardless of gestational age, low birth weight (LBW) was defined as < 2,500 grams [28] and high birth weight (HBW) or macrosomia as ≥ 4,000 grams [26].

### 2.5. Statistical Analysis

Descriptive statistics of the study sample stratified by ethnicity were conducted using Chi-square tests or Fisher Exact tests for categorical variables and *t*-tests for continuous variables. Estimated means and standard errors for total GWG were computed for each ethnic group and by prepregnancy weight status category within ethnic group, adjusting for gestational age at the last prenatal visit. Unadjusted associations of GWG status (below, within, or above IOM-recommended range) with participant characteristics were estimated using contingency tables and Chi-square tests. Adjusted associations of GWG status with participant characteristics were estimated using multinomial logistic regression models (within GWG guidelines as the outcome reference category) to allow for the possibility of associations that violated the proportional odds assumption (e.g., a positive association with both above and below GWG guidelines).

Potential effect modification by ethnicity was examined by stratifying contingency tables of GWG status with participant characteristics by ethnicity and by including interaction terms of ethnicity with other predictors in logistic regression models. Model fit was assessed using the Hosmer-Lemeshow goodness-of-fit Chi-square statistic [29]. Infant outcomes were compared by GWG status for the entire group and by ethnicity using contingency tables, Chi-square tests, and logistic regression. Supplemental analyses included conducting backward elimination in the logistic regression analyses to assess whether results were similar after omitting irrelevant or redundant predictors and performing sensitivity analysis comparing results based on the 1990 IOM GWG guidelines versus the 2009 IOM GWG guidelines.

## 3. Results

The final analytic sample included 571 participants (47% White and 53% Latina). The majority of participants were single (64%) and unemployed (53%) and had public health insurance (64%) (Table 1). Less than half (46%) of women had prepregnancy BMIs within the healthy weight range, a quarter were obese, and more than half (58%) exceeded GWG recommendations at the time of delivery. Compared to White women, Latina women were younger and more likely to be single and unemployed, have public insurance, and have higher parity (*p* values < 0.05). White women had higher prevalence of documented tobacco and alcohol use, were more likely to have a documented psychiatric history, and were more likely to deliver LGA neonates than Latina women (*p* values < 0.05). No other differences by ethnicity were observed. A comparison by prenatal care site revealed that women receiving care in hospital clinics were more likely to be younger, unmarried, unemployed, and nulliparous, have public insurance, have a psychiatric history, and have lower levels of education than those receiving care in private clinics (*p* values < 0.01).

**Figure 1:**
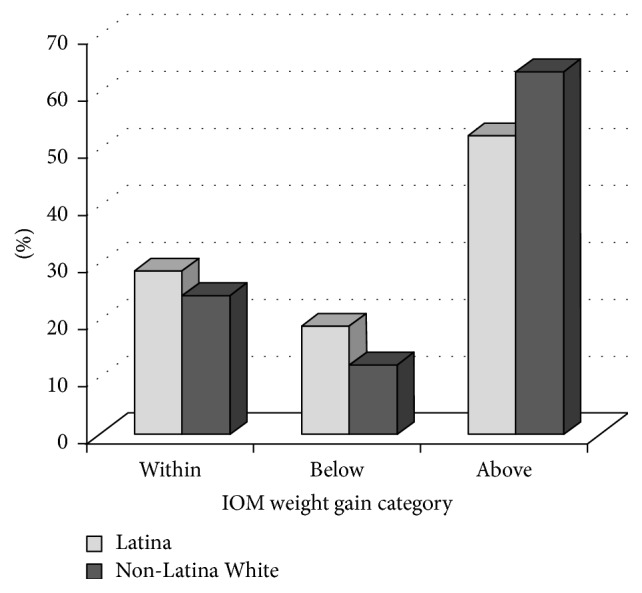
Gestational weight gain status among White non-Latina and Latina women.

**Figure 2:**
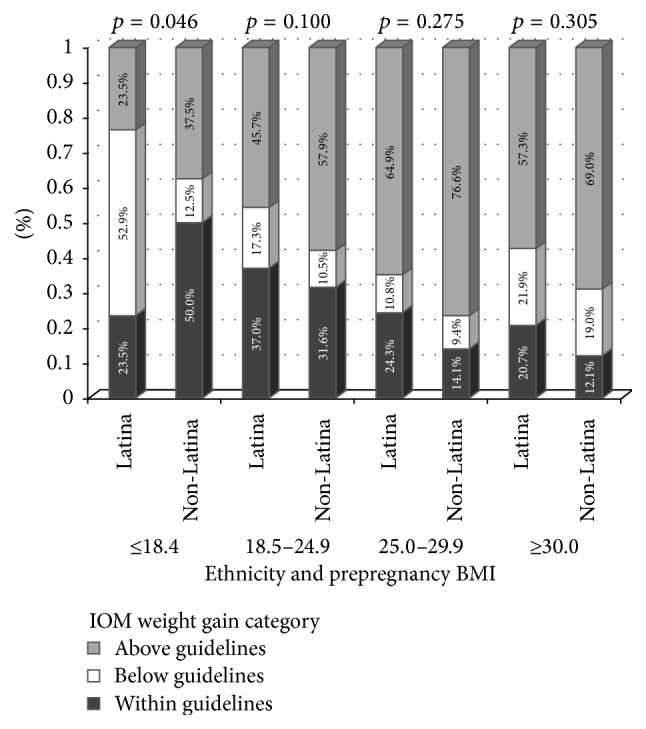
Gestational weight gain status by prepregnancy BMI for White non-Latina and Latina women.

Average GWG adjusted for gestational age at delivery was 36.3 lbs (SE = 0.92) (16.5 kg (SE = 0.42)) for White women and 32.4 lbs (SE = 0.88) (14.7 kg (SE = 0.36)) for Latina women (*p* < 0.0001). Average GWG by prepregnancy weight status category were as follows: 37.9 lbs (SE = 2.3) (17.48 kg (SE = 1.0)) for underweight participants; 36.7 lbs (SE = 0.9) (16.6 kg (SE = 0.4)) for healthy weight participants; 35.3 lbs (SE = 1.2) (35.3 kg (SE = 0.5)) for overweight participants; and 28.0 lbs (SE = 1.2) (12.7 kg (SE = 0.5)) for obese participants. Across prepregnancy weight status categories, adherence to IOM GWG recommendations was poor among both ethnic groups, with only 27% gaining within recommended ranges. Ethnic differences in GWG status at time of delivery for the overall sample were observed, with Latina women less likely to gain in excess than White women (*p* = 0.016) (Figure 1). Latina women were more likely to gain within the IOM-recommended range than White women across all prepregnancy weight status categories, with the exception of the underweight category (among underweight participants, White women were more likely to have GWG within recommended ranges than Latinas) (Figure 2).

Table 2 presents unadjusted associations between demographics, behavioral factors and psychiatric history, and GWG status. GWG status was significantly associated with ethnicity, employment status at pregnancy onset, prepregnancy BMI, and first-trimester GWG (*p* values < 0.05). In logistic regression models, no effect modification by ethnicity was indicated (*p* values for interaction terms > 0.05); thus, results are presented for the entire sample. Multivariable logistic regression models estimating participant characteristics associated with GWG status at time of delivery (Table 3) indicated that odds of above-guideline GWG at time of delivery were greater among prepregnancy overweight and obese women compared to healthy weight women (OR = 3.4, CI = 1.8–6.5; OR = 4.5, CI = 2.3–9.0, resp.) and among those with first-trimester GWG above guidelines compared to those with GWG within guidelines (OR = 4.9, CI = 2.8–8.8). Odds of below-guideline GWG at time of delivery were greater among prepregnancy underweight and obese women compared to healthy weight (OR = 5.3, CI = 1.4–20.2; OR = 3.5, CI = 1.4–8.7, resp.) and among women with first-trimester GWG below guidelines compared to within-guideline GWG (OR = 3.0, CI = 1.3–6.8). Odds of below-guideline GWG were lower among women receiving care at hospital clinics compared to those receiving care from a private provider and among past smokers compared to never smokers (OR = 0.3, CI = 0.1–0.9; OR = 0.3, CI = 0.1–1.0, resp.).

A very small number of adverse events were observed within each ethnic group. Thus, Table 4 presents estimates of associations between GWG status and length of pregnancy (preterm versus full-term) and birth weight parameters for the overall sample. GWG status was unrelated to pregnancy length but was associated with birth size (a higher percentage of SGA in pregnancies with below-guideline GWG and a higher percentage of LGA in pregnancies with above-guideline GWG; *p* values < 0.05). Observed ethnic differences in birth size (Table 1) by which White women were more likely to have LGA neonates and Latina women were more likely to have SGA neonates were not impacted when adjusted for GWG status (data not shown). Supplemental analyses from running more parsimonious models and from sensitivity tests did not yield results that were substantially different from those presented (data not shown).

## 4. Discussion

Findings from this retrospective cohort study provide insights for identifying women at risk for nonadherence to IOM-recommended GWG and for developing targeted interventions. Above-guideline GWG was greater in this cohort (58%) than in previous studies of multiethnic samples (35%–57% in prior studies) [2–4, 7], suggesting that rates of above-guideline GWG may continue to increase, especially among White women. As noted in other populations [2, 7, 30], prepregnancy weight status predicted GWG in this study. Targeting weight prior to pregnancy is desirable but may be unfeasible for numerous reasons, such as lack of pregnancy intentionality. Targeting weight change during pregnancy may be a more feasible window, as a majority of women seek prenatal care during the first-trimester and are motivated to modify health behaviors [31]. To our knowledge, this is the first study to examine first-trimester GWG status as a predictor of GWG status at time of delivery in a multiethnic sample of women, with first-trimester GWG status predicting overall GWG status among non-Latina White and Latina women. Along with other research [22], study findings indicate that the first trimester of pregnancy may be a critical and feasible window to promote healthy GWG and associated maternal and neonatal outcomes; thus, the identification of women who are at elevated risk for below or above GWG guidelines (e.g., prepregnancy underweight and overweight/obese women) and subsequent delivery of targeted interventions for these subgroups during early prenatal care should be emphasized.

For both non-Latina White and Latina women in our study sample, maternal smoking status (previous smoker prior to pregnancy) was associated with lower odds of below-recommended GWG which is consistent with previous research indicating that smoking during pregnancy is related to lower GWG and smoking cessation associated with greater GWG [2, 3, 32, 33]. Between 29% and 70% of women reportedly quit smoking upon becoming pregnant [34]; thus, health care provider attention to smoking history and smoking patterns during pregnancy, with particular focus given to previous or current smokers during early prenatal care, is important to optimize GWG throughout pregnancy.

A larger proportion of SGA infants were born to Latina women than non-Latina White women, with the prevalence of SGA (12.2%) and preterm delivery (13.0%) among Latina women in our sample slightly higher than national estimates for Latina women (9%-10%) [35]. In contrast, a greater proportion of LGA infants were born to White women. We did not find an association between GWG status at time of delivery and pregnancy length as previously found [36]. In addition, we did not find ethnic differences in low or high birth weight, which is in contrast to prior data indicating that Puerto Rican Latinas have some of the highest rates of low birth weight neonates [15] and preterm births [16] in the US. Multiple factors not assessed in this retrospective cohort study (e.g., prior preterm births, gestational diabetes mellitus) may contribute to and account for differences in birth outcomes observed in this study compared to previous studies. In addition, conventional measures of GWG may introduce bias when studying GWG-preterm birth associations [37]. Additional studies with larger, ethnically diverse samples are needed to elucidate predictors driving racial/ethnic disparities in birth weight outcomes.

Study strengths include the sample's ethnic and socioeconomic diversity (i.e., White/Latina women, public/commercial insurance, and hospital clinics/private provider) and inclusion of women who delivered pre- and full-term (previous studies have been limited to women who delivered full-term) [2, 7]. Although no data were available on place of birth, most Latinos in the region where the study was conducted are of Puerto Rican descent, a largely understudied population with considerable health disparities, including infant mortality [38].

Study limitations include the retrospective study design and the use of existing medical record data (with data gathered within the context of clinical activities rather than by trained research staff). However, all providers completed similar maternal and prenatal medical forms, which were routinely filed in the hospital medical record database prior to delivery. Participants' self-reported prepregnancy weight (as opposed to prepregnancy weight measured in a clinical or research setting) was used to determine GWG status. However, the IOM guidelines are based on studies that similarly use self-reported prepregnancy weight [39], and self-reported prepregnancy weight has been found to be highly correlated with clinically measured weight [40–42]. Information available on smoking patterns during pregnancy (i.e., number of cigarettes, quit date) was restricted. Furthermore, smoking status data was collected in the context of the first prenatal appointment and may be subject to social desirability bias and may only reflect smoking status at the first prenatal visit. However, the prevalence of smoking in our sample (19%) is consistent with smoking rates among White [2, 3, 32, 33] and Latina pregnant women [43] in previous studies. Presence of gestational diabetes, shown to be associated with birth weight [44, 45], was not controlled for. Women without a first-trimester prenatal visit and with missing prepregnancy BMI data were excluded from analysis; as systematic biases might exist between women who were or were not missing these data, findings may not be representative of the larger population from which the study sample was drawn. Study findings may not be generalizable to other (non-Puerto Rican) Latino subgroups. Lastly, the study was not adequately powered to examine ethnic differences in pregnancy outcomes by GWG status; thus, results of GWG associated with outcomes of interest by ethnicity are exploratory.

Understanding factors that contribute to inadequate and excessive GWG is critical to the development of interventions that seek to optimize recommended GWG. Additional researches on racial/ethnic differences in the influence of early GWG on GWG and other maternal and neonatal outcomes are needed to guide the development of interventions tailored for socioeconomically and ethnically diverse populations.

## Figures and Tables

**Table 1:** Sample characteristics of overall study sample and by ethnicity (*N* = 571).

Sample characteristics	All women (*n* = 571)		White non-Latina (*n* = 271)		Latina (*n* = 300)		*p* value
	*N*	%	*N*	%	*N*	%	
	Mean	SD	Mean	SD	Mean	SD	
*Demographic factors*							

Age category, *N* (%)							
Age 15–19	127	22.24	39	14.39	88	29.33	<0.001
Age 20–24	155	27.15	64	23.62	91	30.33	
Age 25–29	133	23.29	67	24.72	66	22.00	
Age 30–34	100	17.51	62	22.88	38	12.67	
Age ≥ 35	56	9.81	39	14.39	17	5.67	
Mean (SD)	25.35	6.40	27.09	6.52	23.79	5.88	<0.001

Marital status, *N* (%)							
Divorced	8	1.41	6	2.21	2	0.67	<0.001
Married	199	35.04	129	47.60	70	23.57	
Single	361	63.56	136	50.18	225	75.76	

Employment at onset of pregnancy *N* (%)							
Employed	266	46.83	153	56.67	113	37.92	<0.001
Not employed	302	53.17	117	43.33	185	62.08	

Parity, *N* (%)							
0	233	42.75	114	44.53	119	41.18	0.003
1	160	29.36	86	33.59	74	25.61	
2	89	16.33	39	15.23	50	17.30	
3 or more	63	11.56	17	6.64	46	15.92	

*Behavioral factors*							

Alcohol use, *N* (%)							
No	449	78.91	193	71.22	256	85.91	<0.001
Yes, past	112	19.68	74	27.31	38	12.75	
Yes, this pregnancy	8	1.41	4	1.48	4	1.34	

Tobacco use, *N* (%)							
No	392	68.89	172	63.47	220	73.83	0.016
Yes, past	67	11.78	34	12.55	33	11.07	
Yes, this pregnancy	110	19.33	65	23.99	45	15.10	

Prepregnancy weight categories (body mass index range), *N* (%)							
Underweight (BMI ≤ 18.4)	33	5.78	16	5.90	17	5.67	
Normal weight (BMI 18.5–24.9)	260	45.53	133	49.08	127	42.33	0.315
Overweight (BMI 25.0–29.9)	138	24.17	64	23.62	74	24.67	
Obese (BMI ≥ 30.0)	140	24.52	58	21.40	82	27.33	

*Prenatal care factors*							

Week gestation at the 1st prenatal visit	10.57	3.07	10.53	2.72	10.61	3.36	0.760

Gestational age at the last visit	35.76	5.39	35.68	5.84	35.83	4.96	0.740

Number of prenatal visits	9.78	3.24	9.84	3.36	9.72	3.14	0.656

Gestational age at delivery	38.97	5.87	38.87	6.91	39.06	4.76	0.705

Prenatal care site, *N* (%)							
Private	272	47.64	137	50.55	135	45.00	0.185
Hospital clinic	299	52.36	134	49.45	165	55.00	

Insurance type, *N* (%)							
Commercial/private	201	35.39	139	51.29	62	20.88	<0.001
No insurance/unknown	4	0.70	4	1.48	0	0.00	
Public	363	63.91	128	47.23	235	79.12	

*GWG status with respect to IOM guidelines*							

GWG status at the last prenatal visit, *N* (%)							
Within guidelines	152	26.62	66	24.35	86	28.67	0.016
Below guidelines	90	15.76	33	12.18	57	19.00	
Above guidelines	329	57.62	172	63.47	157	52.33	

GWG status in the 1st trimester, *N* (%)							
Within guidelines	148	26.43	69	26.24	79	26.60	0.537
Above guidelines	256	45.71	126	47.91	130	43.77	
Below guidelines	156	27.86	68	25.86	88	29.63	

*Psychiatric factors*							

Psychiatric history, *N* (%)							
None	445	78.21	203	75.19	242	80.94	0.003
Anxiety	15	2.64	11	4.07	4	1.34	
Depression	86	15.11	38	14.07	48	16.05	
Other	23	4.04	18	6.67	5	1.67	

Psychiatric medications, *N* (%)							
No	539	94.40	251	92.62	288	96.00	0.080
Yes	32	5.60	20	7.38	12	4.00	

*Pregnancy outcomes*							

Length of pregnancy, *N* (%)							
Term delivery	504	88.73	244	90.71	260	86.96	0.158
Preterm delivery	64	11.27	25	9.29	39	13.04	

Birth weight parameters							
SGA	56	10.04	20	7.60	36	12.20	0.012
Normal GA	454	81.36	212	80.61	242	82.03	
LGA	48	8.60	31	11.79	17	5.76	

LBW (<2500 gr)	53	9.45	23	8.68	30	10.14	0.390
Normal BW	456	81.28	213	80.38	243	82.09	
HBW (>4000 gr)	52	9.27	29	10.94	23	7.77	

*p* values are from Chi-square and *t*-tests for ethnic differences.

**Table 2:** Univariate associations of demographic, behavioral, and psychological factors and gestational weight gain status based on 2009 IOM recommendations (*N* = 571).

Category	GWG below guidelines		GWG within guidelines		GWG above guidelines		*p* value
	*N*	%	*N*	%	*N*	%	
Age category							
Age 15–19	26	20.47	26	20.47	75	59.06	0.179
Age 20–24	26	16.77	43	27.74	86	55.48	
Age 25–29	20	15.04	34	25.56	79	59.40	
Age 30–34	14	14.00	35	35.00	51	51.00	
Age ≥ 35	4	7.14	14	25.00	38	67.86	

Ethnicity							
Latina	57	19.00	86	28.67	157	52.33	0.016
White	33	12.18	66	24.35	172	63.47	

Marital status							
Divorced	1	12.50	2	25.00	5	62.50	0.931
Married	29	14.57	57	28.64	113	56.78	
Single	60	16.62	93	25.76	208	57.62	

Employment at onset of pregnancy							
Employed	33	12.41	82	30.83	151	56.77	0.044
Not employed	55	18.21	70	23.18	177	58.61	

Insurance type							
Commercial/private	21	10.45	64	31.84	116	57.71	0.064
No insurance/unknown	1	14.29	2	28.57	4	57.14	
Public	68	18.73	86	23.69	209	57.58	

Obstetric provider							
Private	42	15.44	76	27.94	154	56.62	0.793
Hospital clinic	48	16.05	76	25.42	175	58.53	

Parity							
0	35	15.02	57	24.46	141	60.52	0.913
1	26	16.25	42	26.25	92	57.50	
2	13	14.61	28	31.46	48	53.93	
3 or more	11	17.46	17	26.98	35	55.56	

Prepregnancy BMI							
BMI ≤ 18.4	11	33.33	12	36.36	10	30.30	<0.001
BMI 18.5–24.9	36	13.85	89	34.23	135	51.92	
BMI 25.0–29.9	14	10.14	27	19.57	97	70.29	
BMI ≥ 30.0	29	20.71	24	17.14	87	62.14	

Tobacco use							
No	65	16.58	112	28.57	215	54.85	0.236
Yes, past	8	11.94	18	26.87	41	61.19	
Yes, this pregnancy	17	15.45	21	19.09	72	65.45	

Alcohol use							
No	68	15.14	123	27.39	258	57.46	0.302
Yes, past	21	18.75	23	20.54	68	60.71	
Yes, this pregnancy	1	12.50	4	50.00	3	37.50	

GWG during the 1st trimester							
Above	8	3.13	43	16.80	205	80.08	0.001
Below	61	39.10	53	33.97	42	26.92	
Within	15	10.14	55	37.16	78	52.70	

Psychiatric diagnosis							
None	71	15.96	123	27.64	251	56.40	0.955
Depression	13	15.12	21	24.42	52	60.47	
Anxiety	3	20.00	3	20.00	9	60.00	
Other	3	13.04	5	21.74	15	65.22	

Psychiatric medications							
No	86	15.96	143	26.53	310	57.51	0.871
Yes	4	12.50	9	28.13	19	59.38	

Psychiatric history or medications							
No	70	16.55	117	27.66	236	55.79	0.325
Yes	20	13.51	35	23.65	93	62.84	

*p* value is from a Chi-square test.

**Table 3:** Multivariate analysis of predictors of gestational weight gain status in overall study sample (*N* = 571).

Variable	Adjusted OR for GWG below guidelines		Adjusted OR for GWG above guidelines	
	Odds ratio (95% CI)	*p* value	Odds ratio (95% CI)	*p* value
GWG during the 1st trimester				
Within guidelines	Reference	<0.0001		<0.0001
Above guidelines	0.29 (0.08–1.01)		4.92 (2.75–8.81)	
Below guidelines	3.01 (1.33–6.81)		0.39 (0.21–0.74)	

Prepregnancy BMI				
18.5–24.9	Reference	0.0072	Reference	<0.0001
≤18.4	5.26 (1.37–20.17)		0.19 (0.06–0.58)	
25.0–29.9	0.83 (0.27–2.58)		3.44 (1.82–6.50)	
≥30.0	3.47 (1.38–8.70)		4.55 (2.29–9.04)	

Ethnicity				
White	Reference	0.9446	Reference	0.0069
Latina	0.97 (0.43–2.17)		0.46 (0.26–0.81)	

Tobacco use				
No	Reference	0.0402	Reference	0.2008
Yes, past	0.26 (0.07–0.99)		1.09 (0.49–2.42)	
Yes, this pregnancy	1.77 (0.64–4.84)		2.01 (0.93–4.31)	

Alcohol use				
No	Reference	0.5637	Reference	0.1978
Yes, past	1.69 (0.64–4.49)		1.15 (0.57–2.30)	
Yes, this pregnancy	0.85 (0.05–13.18)		0.15 (0.02–1.29)	

Age group				
25–29	Reference	0.2141	Reference	0.1470
15–19	3.07 (0.87–10.91)		1.50 (0.61–3.67)	
20–24	1.35 (0.45–4.04)		0.83 (0.40–1.74)	
30–34	0.60 (0.20–1.76)		0.52 (0.25–1.08)	
≥35	0.50 (0.10–2.44)		1.18 (0.49–2.86)	

Insurance				
Commercial/private	Reference	0.3929	Reference	0.1126
Public	1.58 (0.55–4.57)		1.75 (0.88–3.50)	

Employment at onset				
Employed	Reference	0.2533	Reference	0.2683
Not employed	1.63 (0.71–3.76)		1.39 (0.77–2.50)	

Parity				
0	Reference	0.9476	Reference	0.5305
1	0.93 (0.38–2.29)		0.94 (0.51–1.74)	
2	1.24 (0.42–3.61)		0.73 (0.34–1.61)	
3 or more	1.28 (0.35–4.59)		0.52 (0.20–1.30)	

Obstetric provider				
Private	Reference	0.0255	Reference	0.3441
Hospital clinic	0.35 (0.14–0.89)		0.75 (0.41–1.37)	

Psychiatric medications				
No	Reference	0.6482	Reference	0.2837
Yes	0.67 (0.12–3.72)		0.51 (0.15–1.73)	

Psychiatric diagnosis				
None	Reference	0.6362	Reference	0.5163
Anxiety	2.08 (0.25–17.06)		1.26 (0.25–6.50)	
Depression	1.14 (0.37–3.52)		1.27 (0.60–2.67)	
Other	3.16 (0.42–23.92)		3.57 (0.66–19.31)	

**Table 4:** Prevalence of selected pregnancy outcomes by gestational weight status in overall study sample (*N* = 571).

Outcomes	Gestational weight gain status		
	Below	Within	Above
Length of pregnancy (*p* = 0.966)^1^			
Term delivery	80 (88.9)	134 (88.2)	290 (89.0)
Preterm delivery	10 (11.1)	18 (11.8)	36 (11.0)

Birth weight (*p* = 0.001)^1^			
SGA	18 (20.7)	15 (10.1)	23 (7.1)
Normal GA	66 (75.9)	124 (83.2)	264 (82.0)
LGA	3 (3.4)	10 (6.7)	35 (10.9)

Birth weight (*p* = 0.061)^1^			
LBW (<2500)	10 (11.5)	18 (12.1)	25 (7.7)
Normal BW	73 (83.9)	122 (81.9)	261 (80.3)
HBW (>4000)	4 (4.6)	9 (6.0)	39 (12.0)

^1^ *p* value for association between GWG status and selected pregnancy outcomes in the entire sample.

GA: gestational age; SGA: small for gestational age; LGA: large for gestational age; BW: birth weight; LBW: low birth weight; HBW: high birth weight.

## Additional Points

*Implications for Practice and/or Policy*. Study findings highlight the importance of identifying and targeting populations at high risk for excessive GWG, particularly in early pregnancy. Emphasizing early prenatal care and facilitating adherence to GWG recommendations in the first trimester are particularly relevant among prepregnancy underweight and overweight/obese women. Within the clinical setting, identifying populations at risk for both above- and below-guideline GWG during early prenatal care is critical for optimizing GWG. Timely targeted interventions are needed for health care providers and practitioners to deliver throughout pregnancy with the ultimate goal of improving maternal and neonatal short- and long-term outcomes.
